# Supplementary figures and images for: Developmental effects of environmental light on male nuptial coloration in Lake Victoria cichlid fish
Source: PeerJ. 2018 Jan 3;6:e4209. doi: 10.7717/peerj.4209 (PMC5756450; doi:10.7717/peerj.4209)

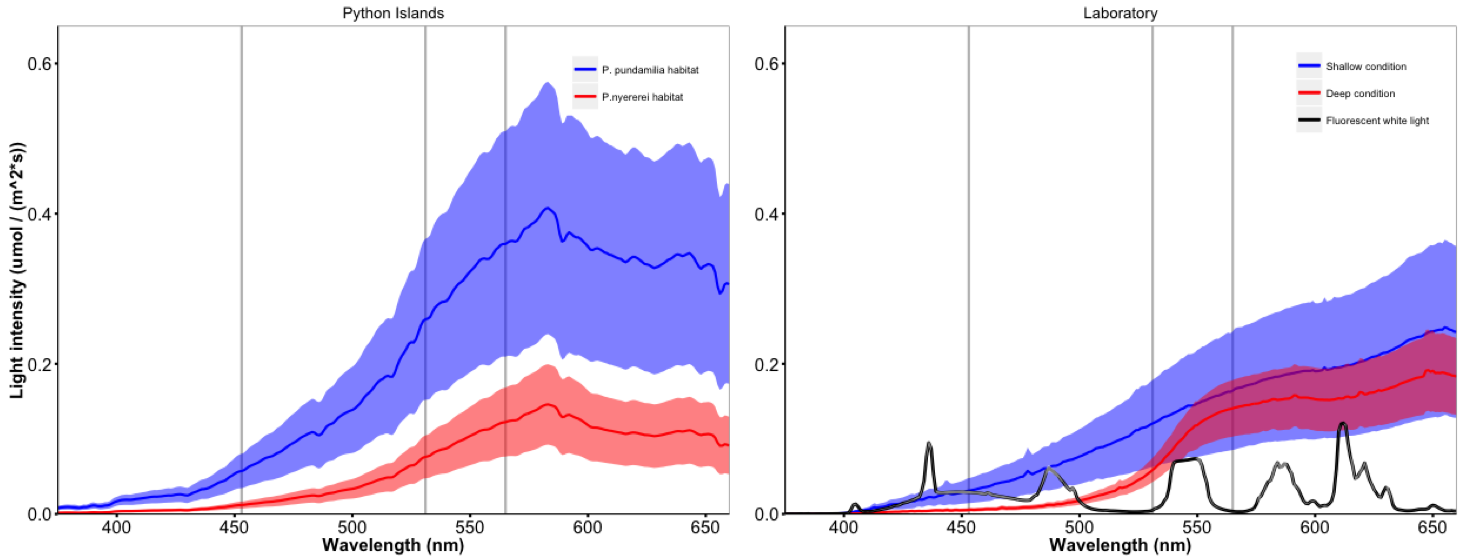

Supplement: Figure S1 — Experimental light environments were created to mimic natural light conditions experienced by P. pundamilia and P. nyererei at Python Islands, Lake Victoria. Vertical lines indicate the peak sensitivities of the three main Pundamilia photoreceptors: SWS2a (453 nm), RH2 (531 nm), LWS (565 nm) (Carleton et al., 2005). [file peerj-06-4209-s001.png]

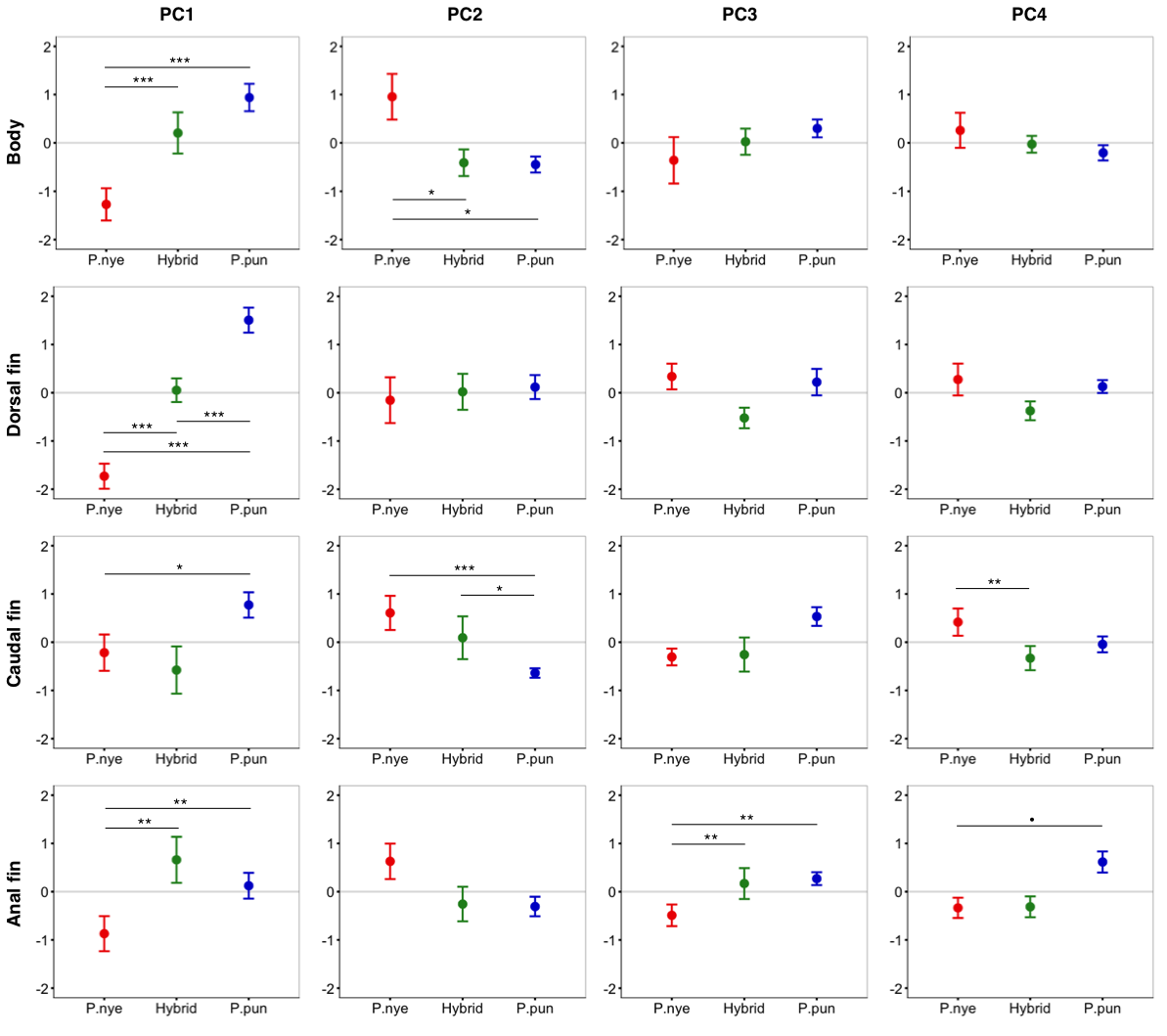

Supplement: Figure S2 — Interspecific differences in coloration, presented as principal components (PC1–PC4), for body and each fin. Error bars represent 95% CI, • indicates P < 0.1, * indicates P < 0.05, ** indicates P < 0.01, *** indicates P < 0.001. [file peerj-06-4209-s002.png]

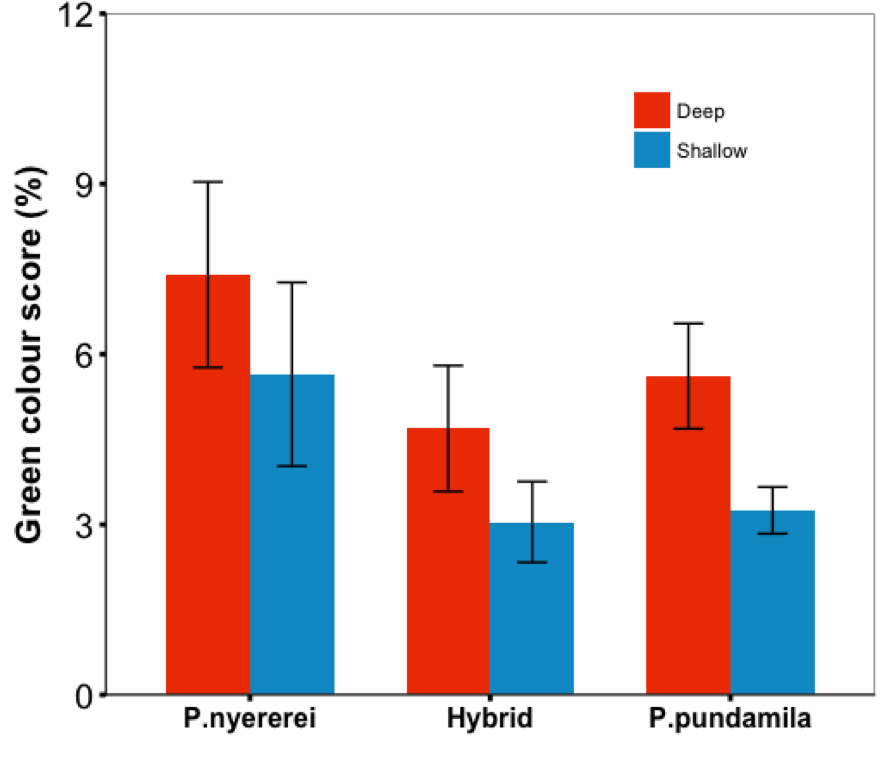

Supplement: Figure S3 — There was no evidence of species-specific response to the light manipulations, as all three species groups exhibited increased green colour in the deep light condition. [file peerj-06-4209-s003.png]

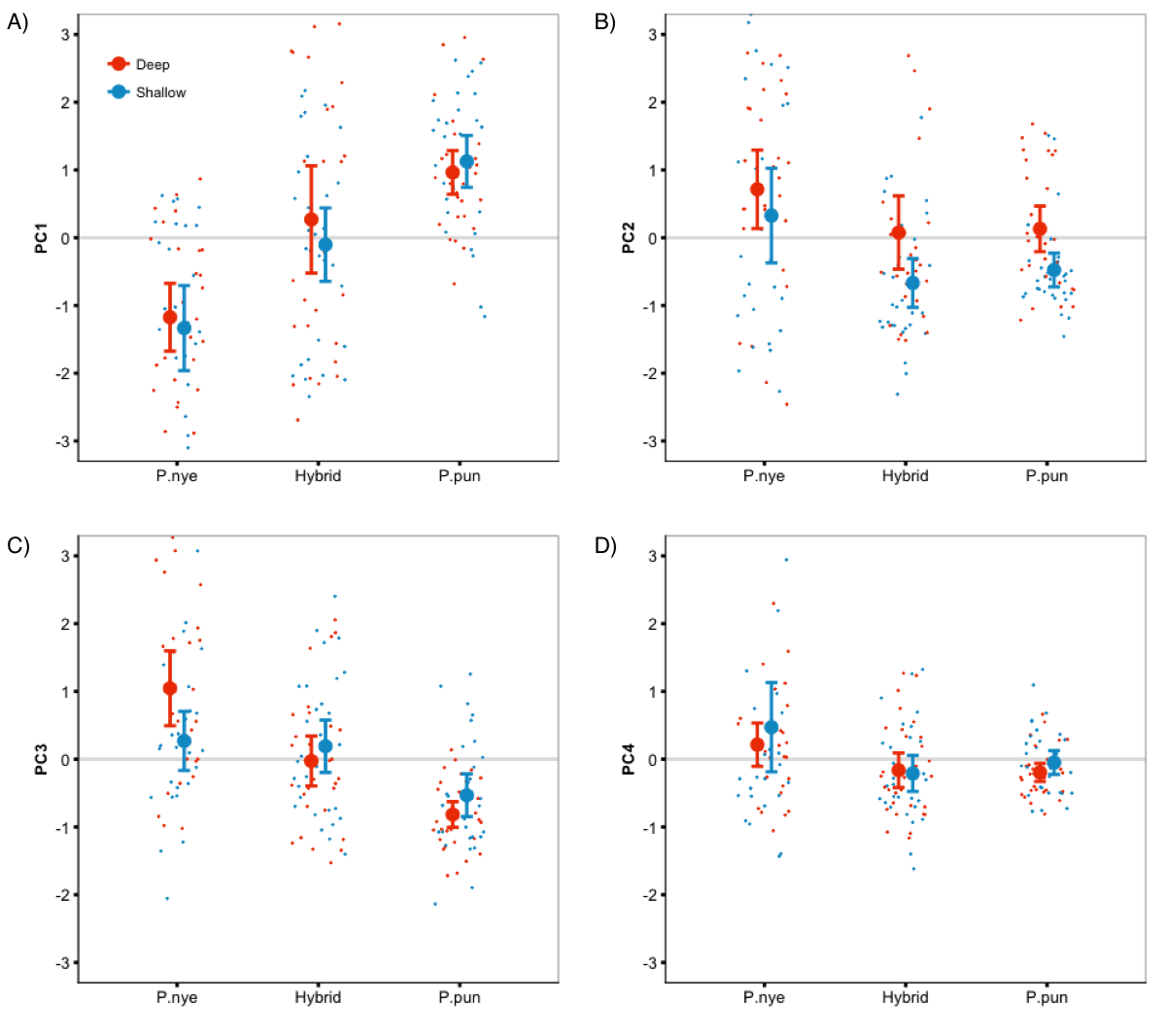

Supplement: Figure S4 — Species-specific coloration was unaffected by our light treatments (the interaction between species and light was non-significant in all analyses). [file peerj-06-4209-s004.png]

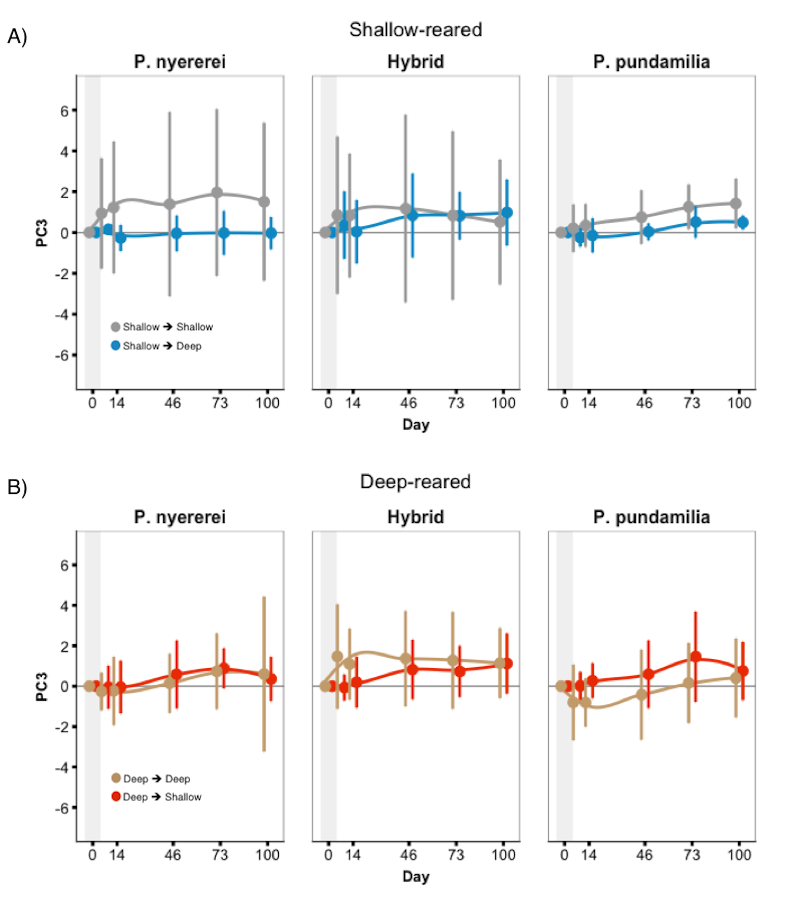

Supplement: Figure S5 — There was a significant three-way interaction between species, treatment, and date for ‘whole fish’ PC3. However, colour differed little between control (SS/DD) and switched fish (SD/DS). Error bars represent 95% CI. [file peerj-06-4209-s005.png]

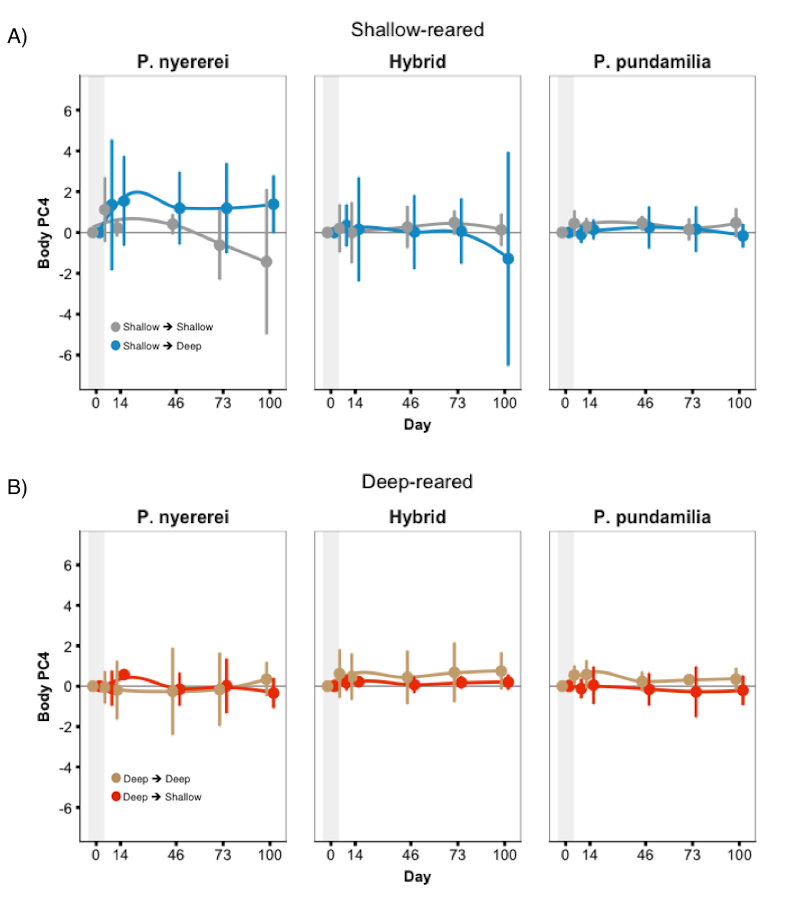

Supplement: Figure S6 — There was a significant three-way interaction between species, treatment, and date for ‘body’ PC4. However, change across the 100-day period differed little between control (SS/DD) and treatment fish (SD/DS). Error bars represent 95% CI. [file peerj-06-4209-s006.png]
