# Supplementary material for: Developmental effects of environmental light on male nuptial coloration in Lake Victoria cichlid fish
Source: PeerJ. 2018 Jan 3;6:e4209. doi: 10.7717/peerj.4209 (PMC5756450; doi:10.7717/peerj.4209)
Supplement: Table S1 — Sample size (males) for each cross, separated by family and by deep (D) and shallow (S) rearing light. Family names are expressed as mother x father, such that ‘PN’ indicates P. pundamilia female x P. nyererei male (F1) and ‘PNPN’ indicates a second generation cross of ‘PN’ female x ‘PN’ male - F1 and F2 hybrids were pooled in the analyses. Superscripted numbers indicate families with the same mothers; superscripted letters indicate families with the same fathers. [file peerj-06-4209-s007.docx]

| ***P. nyererei*** | | | | ***Hybrid*** | | | | ***P. pundamilia*** | | | |
| --- | --- | --- | --- | --- | --- | --- | --- | --- | --- | --- | --- |
| Family | D | S | Gen. | Family | D | S | Gen. | Family | D | S | Gen. |
| NN3 | 1 | 1 | F1 | PN1 | 1 | 1 | F1 | PP9^1^ | 4 | 4 | F1 |
| NN14^a^ | 1 | 1 | F1 | PN8 | 2 | 2 | F1 | PP10^2b^ | 1 | 1 | F1 |
| NN17^a^ | 1 | 1 | F1 | PN10 | 1 | 1 | F1 | PP12^b^ | 1 | 1 | F1 |
| NN18^a^ | 2 | 2 | F1 | NP6 | 1 | 1 | F1 | PP13^2b^ | 1 | 1 | F1 |
| NN19 | 1 | 1 | F1 | NNPP2 | 1 | 1 | F2 | PP14^1^ | 3 | 3 | F1 |
| NN21^a^ | 3 | 3 | F1 | PNPN1 | 1 | 1 | F2 |  |  |  |  |
|  |  |  |  | PNPN3 | 1 | 1 | F2 |  |  |  |  |
|  |  |  |  | PNPN5 | 1 | 1 | F2 |  |  |  |  |
|  |  |  |  | PNNP4 | 1 | 1 | F2 |  |  |  |  |
| *Total* | *9* | *9* |  | *Total* | *10* | *10* |  | *Total* | *10* | *10* |  |
