# Supplementary material for: Developmental effects of environmental light on male nuptial coloration in Lake Victoria cichlid fish
Source: PeerJ. 2018 Jan 3;6:e4209. doi: 10.7717/peerj.4209 (PMC5756450; doi:10.7717/peerj.4209)
Supplement: Table S2 — Sample size (males) for each cross, separated by family and treatment group; for example: ‘DS’ indicates a deep-reared fish that was moved to shallow light. Once again, family names are expressed as mother x father and were pooled in the analyses. Superscripted numbers indicate families with the same mothers; superscripted letters indicate families with the same fathers. [file peerj-06-4209-s008.docx]

| ***P. nyererei*** | | | | | ***Hybrid*** | | | | | ***P. pundamilia*** | | | | |
| --- | --- | --- | --- | --- | --- | --- | --- | --- | --- | --- | --- | --- | --- | --- |
| Family | DD | DS | SS | SD | Family | DD | DS | SS | SD | Family | DD | DS | SS | SD |
| NN14^a^ | 0 | 1 | 0 | 1 | NP6 | 0 | 1 | 0 | 1 | PP9^1^ | 1 | 1 | 1 | 1 |
| NN17^a^ | 0 | 1 | 0 | 1 | PN8 | 0 | 1 | 0 | 1 | PP10^2b^ | 0 | 1 | 0 | 1 |
| NN18^a^ | 1 | 1 | 1 | 1 | PN10 | 1 | 0 | 1 | 0 | PP12^b^ | 1 | 0 | 1 | 0 |
| NN19 | 1 | 0 | 1 | 0 | NNPP2 | 1 | 0 | 1 | 0 | PP13^2b^ | 0 | 1 | 0 | 1 |
| NN21^a^ | 1 | 1 | 1 | 1 | PNNP4 | 0 | 1 | 0 | 1 | PP14^1^ | 1 | 1 | 1 | 1 |
|  |  |  |  |  | PNPN3 | 1 | 0 | 1 | 0 |  |  |  |  |  |
|  |  |  |  |  | PNPN5 | 0 | 1 | 0 | 1 |  |  |  |  |  |
| *Total* | *3* | *4* | *3* | *4* | *Total* | *3* | *4* | *3* | *4* | *Total* | *3* | *4* | *3* | *4* |
