# Supplementary material for: Developmental effects of environmental light on male nuptial coloration in Lake Victoria cichlid fish
Source: PeerJ. 2018 Jan 3;6:e4209. doi: 10.7717/peerj.4209 (PMC5756450; doi:10.7717/peerj.4209)
Supplement: Table S3 — Colour space parameters based on those defined by Selz et al. (2016), modified slightly to accommodate our photography set up, and including ‘green’ and ‘violet’ to cover the entire hue range. Black was defined using the YUV colour space. [file peerj-06-4209-s009.docx]

| **RGB colour space** | | | |
| --- | --- | --- | --- |
|  | **Hue** | **Saturation** | **Brightness** |
| **Red** | 0-18 | 27-255 (10-100%) | 51-254 (20% - 99.9%) |
| **Orange** | 19-26 | 27-255 (10-100%) | 51-254 (20% - 99.9%) |
| **Yellow** | 27-60 | 27-255 (10-100%) | 51-254 (20% - 99.9%) |
| **Green** | 61-114 | 27-255 (10-100%) | 51-254 (20% - 99.9%) |
| **Blue** | 115-170 | 27-255 (10-100%) | 51-254 (20% - 99.9%) |
| **Violet** | 171-194 | 27-255 (10-100%) | 51-254 (20% - 99.9%) |
| **Magenta** | 195-254 | 27-255 (10-100%) | 51-254 (20% - 99.9%) |
|  |  |  |  |
| **YUV colour space** | | | |
|  | **Y** | **U** | **V** |
| **Black** | 0-40 | 0-255 (0-100%) | 0-255 (0-100%) |
