# Supplementary material for: Developmental effects of environmental light on male nuptial coloration in Lake Victoria cichlid fish
Source: PeerJ. 2018 Jan 3;6:e4209. doi: 10.7717/peerj.4209 (PMC5756450; doi:10.7717/peerj.4209)
Supplement: Table S4 — PCA loading matrices from experiment 1, with the cumulative amount of variance accounted for per PC. All PCs were calculated independently for each section. [file peerj-06-4209-s010.docx]

| **Fish** | *PC1* | *PC2* | *PC3* | *PC4* |
| --- | --- | --- | --- | --- |
| *Red* | -0.251 | -0.085 | 0.706 | -0.157 |
| *Orange* | 0.358 | -0.080 | 0.538 | 0.295 |
| *Yellow* | 0.517 | -0.068 | -0.003 | 0.258 |
| *Green* | 0.069 | 0.688 | 0.073 | -0.061 |
| *Blue* | -0.298 | 0.564 | -0.095 | 0.042 |
| *Magenta* | -0.398 | 0.070 | 0.395 | 0.081 |
| *Violet* | -0.334 | -0.031 | -0.111 | 0.886 |
| *Black* | -0.422 | -0.430 | -0.173 | -0.158 |
| *% var.* | 36.4 | 58.0 | 74.6 | 84.6 |

| **Body** | *PC1* | *PC2* | *PC3* | *PC4* |
| --- | --- | --- | --- | --- |
| *Red* | -0.134 | 0.458 | -0.535 | 0.012 |
| *Orange* | 0.441 | 0.142 | -0.232 | 0.409 |
| *Yellow* | 0.554 | 0.039 | -0.031 | 0.186 |
| *Green* | -0.022 | 0.563 | 0.397 | -0.195 |
| *Blue* | -0.353 | 0.353 | 0.391 | 0.119 |
| *Magenta* | -0.293 | 0.340 | -0.513 | 0.067 |
| *Violet* | -0.313 | -0.086 | 0.173 | 0.855 |
| *Black* | -0.414 | -0.451 | -0.237 | -0.105 |
| *% var.* | 32.6 | 55.8 | 75.4 | 86.2 |

| **Dorsal** | *PC1* | *PC2* | *PC3* | *PC4* |
| --- | --- | --- | --- | --- |
| *Red* | -0.491 | 0.222 | -0.068 | 0.006 |
| *Orange* | -0.217 | 0.516 | 0.073 | -0.401 |
| *Yellow* | 0.422 | 0.283 | 0.482 | 0.038 |
| *Green* | 0.420 | 0.043 | 0.433 | 0.145 |
| *Blue* | 0.292 | -0.416 | -0.429 | 0.158 |
| *Magenta* | -0.440 | -0.132 | 0.284 | 0.572 |
| *Violet* | -0.272 | -0.418 | 0.507 | 0.005 |
| *Black* | -0.067 | -0.487 | 0.218 | -0.682 |
| *% var.* | 33.1 | 57.6 | 70.8 | 81.0 |

| **Anal** | *PC1* | *PC2* | *PC3* | *PC4* |
| --- | --- | --- | --- | --- |
| *Red* | 0.357 | -0.313 | 0.074 | -0.608 |
| *Orange* | -0.285 | -0.515 | 0.236 | -0.292 |
| *Yellow* | -0.463 | -0.189 | 0.216 | 0.219 |
| *Green* | -0.392 | 0.341 | -0.142 | -0.316 |
| *Blue* | -0.273 | 0.552 | -0.148 | -0.371 |
| *Magenta* | 0.397 | 0.268 | 0.207 | 0.371 |
| *Violet* | 0.235 | 0.323 | 0.708 | -0.299 |
| *Black* | 0.369 | -0.056 | -0.553 | -0.184 |
| *% var.* | 30.8 | 50.8 | 73.3 | 82.2 |

| **Caudal** | *PC1* | *PC2* | *PC3* | *PC4* |
| --- | --- | --- | --- | --- |
| *Red* | -0.473 | 0.219 | -0.353 | 0.125 |
| *Orange* | 0.313 | -0.019 | -0.661 | -0.194 |
| *Yellow* | 0.478 | -0.265 | 0.367 | -0.204 |
| *Green* | 0.141 | 0.621 | 0.045 | -0.314 |
| *Blue* | 0.043 | 0.652 | 0.174 | -0.162 |
| *Magenta* | -0.308 | -0.057 | 0.453 | -0.345 |
| *Violet* | 0.235 | 0.248 | 0.226 | 0.811 |
| *Black* | -0.527 | -0.085 | 0.119 | 0.054 |
| *% var.* | 31.2 | 54.1 | 67.9 | 78.9 |

| **Fin spots** | *PC1* | *PC2* |
| --- | --- | --- |
| *Red* | -0.200 | -0.890 |
| *Orange* | -0.653 | 0.432 |
| *Yellow* | 0.730 | 0.142 |
| *% var.* | 58.3 | 96.8 |
