# Supplementary material for: Developmental effects of environmental light on male nuptial coloration in Lake Victoria cichlid fish
Source: PeerJ. 2018 Jan 3;6:e4209. doi: 10.7717/peerj.4209 (PMC5756450; doi:10.7717/peerj.4209)
Supplement: Table S5 — PCA loading matrixes from experiment 2, with the cumulative amount of variance accounted for per PC. All PC’s were calculated independently for each section. [file peerj-06-4209-s011.docx]

| **Fish** | *PC1* | *PC2* | *PC3* | *PC4* |
| --- | --- | --- | --- | --- |
| *Red* | -0.302 | -0.137 | 0.688 | -0.206 |
| *Orange* | 0.329 | -0.270 | 0.563 | 0.166 |
| *Yellow* | 0.506 | -0.179 | -0.137 | 0.286 |
| *Green* | -0.112 | -0.598 | -0.210 | -0.357 |
| *Blue* | -0.391 | -0.319 | -0.357 | 0.024 |
| *Magenta* | -0.421 | -0.268 | 0.137 | 0.137 |
| *Violet* | -0.336 | -0.021 | 0.009 | 0.825 |
| *Black* | -0.297 | 0.587 | 0.033 | -0.144 |
| *% var.* | 35.9 | 59.1 | 74.1 | 85.0 |

| **Body** | *PC1* | *PC2* | *PC3* | *PC4* |
| --- | --- | --- | --- | --- |
| *Red* | -0.142 | 0.468 | -0.514 | 0.166 |
| *Orange* | 0.383 | 0.297 | -0.352 | -0.218 |
| *Yellow* | 0.548 | 0.154 | 0.134 | -0.200 |
| *Green* | -0.158 | 0.465 | 0.491 | 0.263 |
| *Blue* | -0.426 | 0.237 | 0.374 | -0.123 |
| *Magenta* | -0.351 | 0.351 | -0.377 | 0.054 |
| *Violet* | -0.310 | 0.019 | -0.014 | -0.881 |
| *Black* | -0.328 | -0.523 | -0.268 | 0.148 |
| *% var.* | 30.6 | 57.0 | 72.1 | 83.5 |

| **Dorsal** | *PC1* | *PC2* | *PC3* | *PC4* |
| --- | --- | --- | --- | --- |
| *Red* | -0.475 | 0.280 | -0.094 | 0.010 |
| *Orange* | -0.053 | 0.619 | -0.105 | 0.149 |
| *Yellow* | 0.505 | 0.193 | 0.314 | -0.124 |
| *Green* | 0.417 | -0.017 | 0.369 | -0.321 |
| *Blue* | 0.145 | -0.494 | -0.055 | 0.693 |
| *Magenta* | -0.451 | -0.009 | 0.496 | -0.054 |
| *Violet* | -0.311 | -0.251 | 0.598 | 0.018 |
| *Black* | -0.147 | -0.441 | -0.373 | -0.612 |
| *% var.* | 32.2 | 53.2 | 67.3 | 78.8 |

| **Anal** | *PC1* | *PC2* | *PC3* | *PC4* |
| --- | --- | --- | --- | --- |
| *Red* | 0.417 | -0.159 | 0.239 | -0.614 |
| *Orange* | -0.370 | -0.284 | -0.301 | -0.578 |
| *Yellow* | -0.500 | -0.021 | -0.312 | 0.159 |
| *Green* | -0.378 | 0.157 | 0.470 | -0.132 |
| *Blue* | -0.292 | 0.327 | 0.606 | 0.015 |
| *Magenta* | 0.255 | 0.547 | -0.300 | 0.144 |
| *Violet* | 0.108 | 0.622 | -0.162 | -0.414 |
| *Black* | 0.367 | -0.276 | 0.222 | 0.231 |
| *% var.* | 31.0 | 48.3 | 63.3 | 73.4 |

| **Caudal** | *PC1* | *PC2* | *PC3* | *PC4* |
| --- | --- | --- | --- | --- |
| *Red* | -0.479 | 0.155 | -0.393 | 0.218 |
| *Orange* | 0.405 | -0.093 | -0.326 | 0.441 |
| *Yellow* | 0.491 | -0.200 | 0.381 | -0.210 |
| *Green* | 0.156 | 0.639 | 0.157 | 0.209 |
| *Blue* | 0.099 | 0.661 | 0.179 | 0.088 |
| *Magenta* | -0.236 | -0.027 | 0.626 | 0.060 |
| *Violet* | 0.071 | 0.284 | -0.310 | -0.804 |
| *Black* | -0.520 | -0.035 | 0.222 | -0.112 |
| *% var.* | 30.2 | 52.2 | 66.7 | 78.6 |

| **Fin spots** | *PC1* | *PC2* |
| --- | --- | --- |
| *Red* | 0.134 | -0.912 |
| *Orange* | 0.676 | 0.368 |
| *Yellow* | -0.749 | 0.174 |
| *% var.* | 59.2 | 97.5 |
