# Supplementary material for: Developmental effects of environmental light on male nuptial coloration in Lake Victoria cichlid fish
Source: PeerJ. 2018 Jan 3;6:e4209. doi: 10.7717/peerj.4209 (PMC5756450; doi:10.7717/peerj.4209)
Supplement: Table S6 — Fish continued to change colour throughout the experiment, as evidenced by the significance of ‘date’ in nearly all analyses. t-values and degrees of freedom presented from linear mixed models; • indicates P < 0.1, * indicates P < 0.05, ** indicates P < 0.01, *** indicates P < 0.001. [file peerj-06-4209-s012.docx]

|  | ***Fish*** | ***Body*** | ***Dorsal fin*** | ***Caudal fin*** | ***Anal fin*** |
| --- | --- | --- | --- | --- | --- |
| ***PC1*** | t(419) = -1.75• | t(419) = -1.55 | t(429) = -3.69*** | t(429) = -4.45*** | t(461) = 1.42 |
| ***PC2*** | t(429) = 0.60 | t(429) = 0.56 | t(429) = 5.38*** | t(429) = 0.48 | t(419) = -2.62** |
| ***PC3*** | t(419) = 5.36*** | t(419) = -2.73** | t(429) = -2.72** | t(429) = -2.34* | t(461) = 2.06* |
| ***PC4*** | t(419) = 0.53 | t(419) = -2.07* | t(429) = -4.09*** | t(429) = 4.74*** | t(429) = -4.44*** |
